# The importance of the one carbon cycle nutritional support in human male fertility: a preliminary clinical report

**DOI:** 10.1186/1477-7827-12-71

**Published:** 2014-07-29

**Authors:** Maurizio Dattilo, Dominique Cornet, Edouard Amar, Marc Cohen, Yves Menezo

**Affiliations:** 1 Parthenogen, Via F. Pelli 1, Lugano 6900, Switzerland; 2 10 rue Jean Richepin, Paris 69016, France; 3 Cabinet d’Andrologie, 17 avenue Victor Hugo, Paris 75116, France; 4 Procrelys Association de recherche en Infertilité, Lyon 69008, France; 5 Laboratoire Clément, 17 avenue d’Eylau, Paris 75016, France

**Keywords:** One carbon cycle, Methylation, Homocysteine, Sperm fertilizing ability, Oxidative damage

## Abstract

**Background:**

Sperm chromatin structure is often impaired; mainly due to oxidative damage. Antioxidant treatments do not consistently produce fertility improvements and, when given at high doses, they might block essential oxidative processes such as chromatin compaction. This study was intended to assess the effect on male sub-fertility of a pure one carbon cycle nutritional support without strong antioxidants.

**Methods:**

Male partners of couples resistant to at least 2 assisted reproductive technology (ART) attempts, with no evidence of organic causes of infertility and with either DNA fragmentation index (DFI) measured by Terminal deoxynucleotidyl transferase dUTP Nick End Labeling (TUNEL) or nuclear decondensation index (SDI) measured by aniline blue staining exceeding 20%, were invited to take part in a trial of a nutritional support in preparation for a further ART attempt. The treatment consisted of a combination of B vitamins, zinc, a proprietary opuntia fig extract and small amounts of N-acetyl-cysteine and Vitamin E (Condensyl™), all effectors of the one carbon cycle.

**Results:**

84 patients were enrolled, they took 1 or 2 Condensyl™ tablets per day for 2 to 12 months. Positive response rates were 64.3% for SDI, 71.4% for DFI and 47.6% for both SDI and DFI. Eighteen couples (21%) experienced a spontaneous pregnancy before the planned ART cycle, all ended with a live birth. The remaining 66 couples underwent a new ART attempt (4 IUI; 18 IVF; 44 ICSI) resulting in 22 further clinical pregnancies and 15 live births. The clinical pregnancy rate (CPR) and the live birth rate (LBR) were 47.6% and 39.3% respectively. The full responders, i.e. the 40 patients achieving an improvement of both SDI and DFI, reported a CPR of 70% and a LBR of 57.5% (p < 0.001).

**Conclusions:**

Nutritional support of the one carbon cycle without strong antioxidants improves both the SDI and the DFI in ART resistant male partners and results in high pregnancy rates suggesting a positive effect on their fertility potential.

## Background

The role of the male factor in couples’ infertility is difficult to quantify as it can be masked by the variable fertility of the female partner. However, according to the latest guidelines of the European Association of Urology [1], a male-infertility-associated factor is found together with abnormal semen parameters in 50% of involuntarily childless couples; in 30-40% of cases no male-infertility-associated factor is found (idiopathic male infertility).

A retrospective and descriptive study of 35 year old men using data registered by Fivnat (France) reported a decrease of the average sperm concentration from 73.6 million/ml in 1989 to 49.9 million/ml in 2005 [2], strongly suggesting that the changing environment significantly affect male fertility. Availability of diagnostic aids to assess oxidative damage, particularly sperm DNA fragmentation Index (DFI) and Sperm chromatin Decondensation Index (SDI), has given rise to great interest in the causative mechanisms and led to the introduction of a variety of interventions aimed at the correction of the oxidative imbalance. In the past decade about 20 published clinical trials confirmed that oral antioxidants exert a positive effect on sperm oxidative stress; however, the majority of these studies were small, heterogeneous and did not generate sufficient data to confirm whether these improvements result in improved secondary outcomes [3]. Nevertheless, the latest available Cochrane review [4] concluded that antioxidant supplementation in sub-fertile males may improve the outcomes of live birth and pregnancy rate for sub-fertile couples undergoing Assisted Reproductive Technologies (ART): based on 20 live births from a total of 214 couples in three studies.

On the other hand, two recent clinical reviews [3,5] concerning antioxidant treatments for male infertility warned of the risks of depleting essential physiological reactive oxygen species (ROS), potentially leading to “reductive stress” [3] which can negatively affect capacitation and the acrosome reaction [5] when high doses of powerful antioxidant combinations are used. These substances have the potential to interfere with the essential process of sperm chromatin compaction that is largely based on the formation of disulfide bridges between the -SH groups of the cysteine-enriched protamines [6]. This is a mild oxidative reaction that could be impaired by “reductive stress”, as shown for Vitamin C in vitro [7]. Treatment with powerful antioxidants, although improving some measures of oxidative damage, e.g. DFI, may result in the worsening of the SDI – a situation that has been observed in a clinical setting [8]. An alternative approach, based upon the body’s own homeostatic system, that minimises the unwanted actions whilst maximizing the desired actions should be considered.

An oxidative attack could be neutralized by the endogenous metabolism, where the universal reducing agent is reduced glutathione (GSH). GSH synthesis is largely based on the transsulphuration of homocysteine (Hcy) formed within the so-called one carbon cycle - a ubiquitous biochemical pathway.

**Figure 1:**
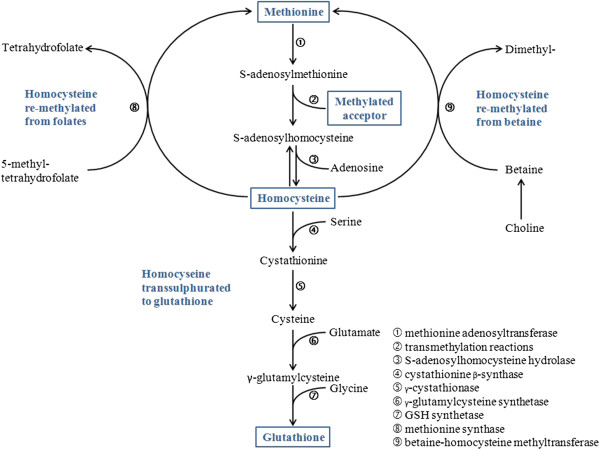
**The one carbon cycle and the transsulphuration pathway.** The one carbon cycle (upper part) provides activated methyl groups for transmethylation reactions: methionine is the carbon unit donor, being activated by adenosylation to SAMe. Release of the carbon unit to any acceptor generates S-adenosyl-homocysteine and then Hcy. Hcy is subsequently remethylated (recycled) to methionine. Alternatively, Hcy may enter the transsulphuration pathway (lower part) by forming a complex with serine by the highly regulated cystathionine β-synthase (4), leading to the synthesis of GSH.

When a molecule is to be elongated by the addition of a methyl unit a molecule of homocysteine is formed in almost all instances, leading to a huge build up of it (Figure 1). Homocysteine subsequently undergoes either a remethylation to methionine and activation to S-adenosyl-methionine (SAMe) or transulphuration to GSH “on-demand”. The rate of Hcy transulfuration to GSH is redox-regulated as the key enzyme, cystathionine-beta-synthase (CBS), contains a heme cofactor that functions as a redox sensor: once an oxidative imbalance occurs the heme is oxidized from the ferrous (Fe^++^) to ferric (Fe^+++^) state which activates the enzyme [9]. The greater the oxidative load, the greater the activation of GSH synthesis. Conversely, CBS undergoes allosteric activation by SAMe, increasing its activity by 2.5-5 fold [10]. In summary, GSH synthesis is triggered by the oxidative load but is effective only as long as SAMe is available, i.e. as long as an adequate dietary intake of folates and other Group B vitamins is in place.

Hcy is at the crossroads of two major metabolic pathways: GSH synthesis that regulates the redox balance and trans-methylations that regulates gene expression (imprinting) and cell growth. Hcy is also an inhibitor of the methylation processes; it has an extremely negative effect on spermatogenesis quality [11] and its concentration in the ejaculate is inversely correlated with fertility outcome [12]. The same observations can be made for oogenesis [13]. Failures in these pathways will decrease endogenous glutathione and increase free homocysteine, with knock-on effects on the related methylation processes. Tailored nutritional support might be able to activate such pathways, strengthening antioxidant defences whilst supporting tissue growth and differentiation leading to an improved sperm maturation process.

A suitable oral nutritional support intended to feed and activate the one carbon cycle and the transsulphuration pathway should contain a full range of group B vitamins, namely B2, B3, B6, B12, and B9 mandatory for recycling homocysteine. N-Acetyl-Cysteine and/or L-cystine, the only orally bioavailable precursors for the synthesis of GSH, and Zinc are also important for homocysteine recycling. Zinc is as an essential co-factor for two key enzymes, dihydrofolate reductase and methionine synthase, and does not have any body reservoirs so that the circulating level is largely dependent on daily intake.

Our aim was to test the efficacy of this combination of substances in improving the fertility of ART-resistant male partners who were selected on the basis of elevated DFI and SDI. The combination (Condensyl™) also contained small amounts of vitamin E, quercetin and betalains of natural origin intended to provide some protection to the cell membranes from excessive peroxidation.

## Methods

### Patients

The male partners of couples with at least 2 previous ART failures and willing to undertake a further attempt were screened for sperm chromatin integrity at 3 ART clinics in France between August 2010 and March 2013. Organic causes of male infertility were carefully excluded. DFI was measured by Terminal deoxynucleotidyl transferase dUTP Nick End Labeling (TUNEL) and SDI was measured by aniline blue staining at the reference laboratories of the concerned clinics according to the original methods [14,15]. TUNEL was selected because it can be performed in situ and in case of low sperm counts it has a good correspondence to flow cytometry [16]. Chromatin decondensation was tested by aniline blue staining because it has better specificity than the alternative test, chromomycin A3, which overlaps to some extent with TUNEL [17].

Patients who had either a DFI or SDI exceeding the 20%, irrespective of their spermiogram, were offered nutritional support in preparation of a new ART procedure. A cut-off value for TUNEL-DFI higher than 20% was selected based on our clinical experience and on published evidence [18]. There is good consensus that a high DFI is a marker for increased sperm DNA damage associated with decreased embryo quality, decreased pregnancy rates and higher rates of spontaneous miscarriage [19]. We also selected patients based on a high aniline blue-SDI which, in our clinical experience, is a strong predictor of reproductive problems. Previous studies using a SDI cut-off value of 10% [20,21] or 15% [22] showed only a discrete predictive value so we used a higher inclusion value of 20%, aiming to be more selective.

### Intervention

This was an exploratory study in patients who had already experienced repeated ART failures so excessive study procedures and major changes in clinical management were not ethically justified. The treatment was offered to all the recruited patients without including a control group. For the same reason the intervention was limited to the offer of nutritional support to be taken according to the product labeling, which is one or two tablet per day. Pre-existing dietary habits as well as other possible environmental factors were not investigated but patients were instructed note and report any major changes occurring during the study period. Sperm sampling and the evaluation of DFI and SDI was conducted within the standard practice at the concerned centers and did not require any procedural adjustment. Sperm sampling and testing was performed at inclusion, as part of the standard work-up, and at the time of the ART cycle. Patients enjoying a spontaneous pregnancy were immediately asked for a sperm donation for research purposes.

The nutritional support consisted of a proprietary extract of *opuntia* fig fruits (100 mg) delivering tailored amounts of quercetin (0.05 mg) and betalain (0.001 mg) plus a mix of Group B vitamins: B2 (1.4 mg), B3 (16 mg), B6 (1.4 mg), B9 (400 μg), B12 (2.5 μg). The administered product also contained zinc (12.5 mg) and small doses of N-acetyl-cysteine (250 mg) and vitamin E (12 mg) (Condensyl™). All the substances were dosed within the Recommended Daily Allowance in the EU. The prescribed starting dose was 1 tablet per day and patients were informed that taking a larger dose of 2 tablets daily had the potential to produce greater benefits but that it was not scientifically proven. Targeted treatment duration was 4 months and was dependent on the patient’s compliance and to the actual planning of the following ART cycle. ART was performed by IUI, IVF or ICSI balancing the clinical indication with the couple’s willingness.

### Statistics

This was an exploratory study, the group was not statistically sized. In addition to basic descriptive statistics (mean, range and standard deviation as appropriate), statistical analysis was performed using IBM SPSS statistics on a personal computer.

For continuous variables we used the Wilcoxon test or the Mann–Whitney test. The Wilcoxon test was used to compare two continuous variables in the same group of patients. The Mann–Whitney test was used to compare continuous variables in two independent groups. In both cases, the null hypothesis was that the values are the same and the alternative hypothesis was that the values are different. A p value < 0.05 was assumed as statistically significant.

The independence of two categorical variables was tested by the independence Chi^2^ test, also known as the test of homogeneity. The null hypothesis was that the two variables are independent, the alternative hypothesis corresponds to the variables having an association or relationship. A p value < 0.05 was assumed as statistically significant.

## Results

A total of 84 patients fulfilled the entry criteria and gave consent for the nutritional intervention. Their demographic characteristics are summarized in Table 1. In 28 cases (33%) there was an associated female factor: ovulation disorders (n = 12), low anti-mullerian hormone (n = 9), polycystic ovaries with or without ovulation disorders (n = 7). The majority of the patients were oligoasthenospermic (61%) or asthenospermic (17%) whereas about 1 in 4 (23%) was normospermic.

**Table 1:** Demographic characteristics of patients and their female partners

	**Patients**	**Female partners**
*Mean age, years (range, SD)*	37 (25–63, 5.5)	35 (25–44, 4.4)
*Normospermic, n (%)*	19 (23%)	
*Asthenospermic, n (%)*	14 (17%)	
*Oligoasthenospermic, n (%)*	51 (61%)	
*Female factor, n (%)*		28 (33%)
*Previous ART cycles (mean n, range)*	2.4 (2–6)	

The average treatment duration was 130 days (range 59–365 days). Fifty-nine patients reported that they took 1 tablet per day, with 5 of them spontaneously increasing it to 2 tablets per day during the treatment period. The remaining 25 patients stated that they took 2 tablets per day from the outset. The majority of the patients were not able to report on the amount of missed doses so that the actual compliance to the treatment could not be assessed. None of the patients reported a major change in the dietary habits or environmental exposure during the study period.

All patients were available for post-treatment DFI and SDI evaluation, which occurred either at the time of the new ART or immediately after the detection of a spontaneous pregnancy, as applicable. The average DFI significantly decreased from 29.7% to 23.1% (p < 0.001) and the average SDI significantly decreased from to 40.1% to 36.3% (p < 0.001). The responder rates (any index decrease) were 71.4% for DFI, 64.3% for SDI and 47.6% for both DFI and SDI (Table 2). Male partners of women with a female factor showed no improvement of either their DFI (+0.8%) or SDI (+4.3%).

**Table 2:** Responder rates and their DFI and SDI values before and after the treatment, mean values (standard deviation)

**Groups**	**n (%)**	**DFI**			**SDI**		
		**Pre**	**Post**	**% change**	**Pre**	**Post**	**% change**
*All patients*	84 (100)	29.7%	23.1%	**−6.6%** (14,16)*	40.1%	36.3%	**−3.8%** (13.87)*
*DFI responders*	60 (71%)	33.0%	20.0%	−13.0% (10.66)	38.5%	34.6%	−3.9% (13.64)
*SDI responders*	54 (64%)	28.5%	21.4%	−7.0% (14.17)	42.0%	30.2%	−11.8% (8.88)
*DFI and SDI responders*	40 (48%)	31.8%	18.8%	−13.1% (10.74)	40.2%	28.9%	−11.4% (8.40)

*p < 0.001, Wilcoxon test. Statistically significant differences in bold.

Treatment did not modify the total sperm count (p = 0.69), fast motility (p = 0.52) and normal morphology (p = 0.1) rates. Normospermic patients had a lower average DFI at inclusion (p = 0.02). There were no differences in normospermic patients vs astheno- and oligoastheno-spermic patients in the percent decrease of DFI (−3.1 *vs −*7.7%, p = 0.19) and SDI (−4.3 *vs −*3.6%, p = 0.6) or in the pregnancy rate (LBR 47.4 *vs* 28.6%, p = 0.41).

Pregnancy rates are summarized in Table 3. In all 40 clinical pregnancies were attained, 33 of them ending with a live birth (1 medical abortion, 4 spontaneous losses, 1 lost at follow-up while ongoing, 1 still ongoing). Eighteen couples (21%) experienced a spontaneous pregnancy during the treatment, all of them ending with a live birth. The remaining 66 couples underwent a new ART attempt (4 IUI; 18 IVF; 44 ICSI), which resulted in 22 further clinical pregnancies, resulting in 15 live births. The overall clinical pregnancy rate (CPR) and live birth rate (LBR) were 47.6% and 39.3% respectively. The 40 patients who showed improvements in both indices obtained 28 pregnancies (CPR = 70%) and 23 live births (LBR = 57.5% - p < 0.000 vs patients not improving both indexes). Patients improving SDI alone reported a higher CPR (p = 0.000) and LBR (p = 0.000), those improving DFI alone reported a higher CPR (p = 0.032). No pregnancies occurred in 10 patients failing to achieve any index improvement. Only 2 pregnancies (1 live birth) were recorded out of 28 couples carrying a female factor compared with 38 pregnancies (32 live births) out of 56 couples without an associated female problem (Table 4).

**Table 3:** **Pregnancy rates in 84 ART-resistant patients treated with Condensyl™**

**Categories**	**n**	**Clinical pregnancies**			**Live births**		
		**n**	**Rate**	**p**	**n**	**Rate**	**p**
*All patients*	84	40	47.6%		33	39.3%	
*Pts improving both DFI and SDI*	40	28	70.0%	**0.000**	23	57.5%	**0.001**
*Pts improving SDI*	54	35	64.8%	**0.000**	30	55.6%	**0.000**
*Pts improving DFI*	60	33	55.0%	**0.032**	26	43.3%	0.23
*Pts not improving DFI nor SDI*	10	0	0.0%	**0.001**	0	0.0%	**0.007**

Independence CHI^2^ test. Statistically significant differences in bold.

**Table 4:** DFI and SDI response and pregnancy rates according to the presence of any female factor

**Groups**	**n**	**DFI**				**SDI**				**Pregnancies**	
		**Pre**	**Post**	**% change**	P*	**Pre**	**Post**	**% change**	P*	**CPR (n)**	**LBR (n)**
*Female factor YES*	28	25.1%	25.9%	0.8%	0.145	41.6%	45.8%	4.3%	0.732	4.1% (2)	2.0% (1)
*Female factor NO*	56	32.1%	21.7%	−10.4%	**0.000**	39.4%	31.6%	−7.8%	**0.000**	67.9% (38)	57.1% (32)
P**				**0.000**				**0.000**			
P***										**0.000**	**0.000**

*Wilcoxon test; **Mann–Whitney test; ***Independence CHI^2^ test. Statistically significant differences in bold.

Duration of treatment had no effect on pregnancy rates: patients treated for 3 months or less (n = 47) had a CPR of 48.9% and a LBR of 40.4% compared with 45.9% and 37.8% (ns) in those with a longer treatment term (n = 37). It is to be noted that the study was not designed to detect a treatment duration effect on pregnancy rates because, in most instances, short duration of treatment was due to the early occurrence of a spontaneous pregnancy (9/19 live births in the short treatment subgroup). In addition, there was no significant dose effect of treatment on the clinical response: patients taking 1 tablet per day (n = 54) recorded a CPR of 46.3% and a LBR of 42.6% compared with 50.0% and 33.3% (ns) in those taking more than 1 tablet per day (n = 30). However, these data are biased by the lack of a reliable control on the actual administration of the prescribed doses.

It was not possible to define DFI and/or SDI cut-off values predictive of a successful pregnancy. However, patients achieving a pregnancy (n = 40), those experiencing a spontaneous pregnancy (n = 18) and those achieving an ART pregnancy (n = 22) all had a lower post-treatment SDI due to a larger SDI decrease (p < 0.001). The subgroup of patients achieving an ART pregnancy also had a larger decrease of DFI which was of borderline significance (p = 0.046). DFI and SDI response versus the occurrence of a clinical pregnancy is detailed in Table 5.

**Table 5:** DFI and SDI response according to the occurrence of a clinical pregnancy, mean values (standard deviation)

**Groups**	**n (%)**	**DFI**				**SDI**			
		**Pre**	**Post**	**% change**	**p**	**Pre**	**Post**	**% change**	**p**
**Any pregnancy**									
YES	40 (47.6)	29.4%	20.1%	−9.3% (13.61)	0.168	40.6%	29.3%	−11.3% (12.81)	**0.000**
*NO*	44 (52.4)	30.1%	25.9%	−4.2% (14.38)		39.6%	42.6%	3.0% (11.10)	
***Spontaneous pregnancy***									
*YES*	18 (21.5)	23.2%	18.4%	−4.8% (9.38)	0.571	44.8%	29.8%	−15.0% (11.11)	**0.000**
*NO*	66 (78.5)	31.5%	24.4%	−7.2% (15.23)		38.8%	38.0%	−0.7% (13.00)	
***ART pregnancy***									
*YES*	22 (33)	34.4%	21.4%	−13.0% (15.52)	**0.046**	37.2%	29.0%	−8.2% (13.54)	**0.001**
*NO*	44 (67)	30.1%	25.9%	−4.2% (14.38)		39.6%	42.6%	3.0% (11.10)	

Mann–Whitney test. Statistically significant differences in bold.

## Discussion

The present study reports a CPR and a LBR of 47.6% and 39.3% in ART-resistant couples carrying an idiopathic male factor, with or without an associated female factor, following nutritional intervention to support the one carbon cycle. It is noteworthy that more than half of all live births (18 out of 33) were achieved spontaneously, i.e. during the run-up to a planned ART cycle.

The study was not controlled, therefore no conclusions on the reported pregnancy rates can be drawn. However, a recent follow-up study on another French population [23] reported a cumulative chance of achieving a pregnancy for couples referring for a male factor of 60% after a 9-year follow-up (year 2000–2008). The birth rate achieved by either ART or natural conception was 45% at 9 years. In the same study, the cumulative birth rate at 2 years, which is far longer than the average period of follow-up in our study, was 28%: 16% from ART and 12% from natural pregnancy. Furthermore, the reported follow-up study included patients at their first referral and female factors had been excluded whereas we included patients selected for proven resistance to ART, irrespective of the presence of a female factor. A comparison of the outcomes from the two studies supports the idea that fertility improvements might have been gained with nutritional intervention.

The nutritional support resulted in a significant decrease of both DFI and SDI, although the mean average magnitude of the decrease was not very high and a possible spontaneous regression to the mean value might apply. However this is unlikely as, in the absence of any therapeutic intervention, DFI and SDI are much more stable over time than the classical semen parameters [24-26]. Moreover, in index response subgroups the magnitude of the index decreases was large and the association with the reproductive outcomes highly significant. In particular, the SDI decrease exerted a very robust association with the occurrence of pregnancies, both spontaneous and following an ART cycle, whereas the DFI decrease showed a borderline correlation with the ART pregnancies.

In contrast with other male infertility studies, we enrolled our patients irrespective of the presence of a female factor, which might better reflect the expected efficacy in clinical practice. The CPR and LBR in the subgroup with an associated female factor were only 4.2% and 2% compared with 67.9% and 57.1% in couples without a known female problem (Table 4). This may indicate that improvements in sperm quality may be of little help as long as a female problem is in place. However, the male partners of these couples also failed completely to improve their fragmentation and decondensation indices which is, by definition, independent of the female factor and was, most likely, due to very poor treatment compliance. For this reason, the outcomes from this study should not be grounds for not treating sperm defects if a female problem also exists.

Besides confirming the role of the one carbon cycle in male fertility and the potential benefit from nutritional support, the present study is exploratory in nature and design; it does not provide fundamental guidance regarding dose, length of treatment and the target values of the various indices.

The amount of nutritional support taken by the patients was variable and we could not assess the actual number of missed doses. The actual dose of treatment taken is the main variable for any treatment that is left in the patient’s control; in the absence of recurring trigger symptoms may lead to the patients forgetting to take their medication. Patient compliance motivation is also a major variable - indirectly suggested by the lack of indices improvement in male partners of women with a female factor. In clinical practice we would expect to see greater variability in compliance, which remains a main constraint of any nutritional treatment.

The variable duration of intervention, ranging from 2–12 months, was another weakness of the study. In most instances the short duration of treatment was due to the occurrence of a spontaneous pregnancy whereas longer durations were mainly associated with the patient’s willingness and ability to accommodate the date of ART in their lives. So there was little hope to discern any correlation between treatment duration and reproductive outcome. Nevertheless, in our clinical experience some patients may require a longer treatment to achieve a reduction of SDI. In one Condensyl treated patient (not included here) with a starting SDI of 42%, a first increase up to 52% was observed. Then a progressive decline, down to 13% occurred after 9 months of treatment, ending with a spontaneous ongoing pregnancy (Tosti E, 2010 unpublished results).

The DFI cut-off value we used as the inclusion criterion, >20%, confirmed to mark a worse prognosis. Indeed, the 24 patients with a baseline DFI < 20% recorded a CPR of 62.5% and a LBR of 54.2% compared with 41.7% and 33.3% in those with a higher baseline DFI, which did not reach statistical significance possibly due to the small sample size. It is to be noted that the 15 patients achieving a clinical pregnancy (13 live births) out of 24 with a baseline DFI <20% had an elevated baseline SDI (45.5%) and enjoyed a deep SDI decrease (−16.2%). Moreover, 9 out of these clinical pregnancies and live births where achieved spontaneously. Conversely, the 9 patients from this subgroup not achieving a clinical pregnancy, who also had a high baseline SDI (41.6%), failed at improving their decondensation rate (+6.3%), which may explain the negative outcome. In addition, 8 out of these 9 patients had an associated female factor, i.e. they are among those strongly suspected of poor compliance. In spite of the very small sample, these data taken together seem to identify a subgroup of infertile patients, those referring with low DFI and high SDI, who have a very high chance of benefiting from the tested nutritional support and this group should be tested in larger prospective trials. Only 4 patients (3 live births) had a baseline SDI below the 20% inclusion cut-off value, thus the sample is too small to allow similar speculation.

The target levels of DFI and SDI marking a positive outcome also remain unaddressed as we did not identify cut-off values. This is likely due to the small sample size, but other mechanisms might apply. Eight out of 18 spontaneous pregnancies recorded in this study occurred in patients whose final SDI was still above the critical level of 30% but all of them had enjoyed a relative decrease greater than 15%. The same trend applied, with less evidence, to the changes in DFI.

In any case it is clear that, in our model, SDI exerted a far greater effect on pregnancies than DFI (Table 5). Indeed, the DNA damage related to the DFI increase can be repaired within the zygote whose DNA repair activity is, by definition, of maternal origin [27]. Thus the final effect of sperm fragmentation level depends on the variable DNA repair ability of the fertilized oocyte, i.e. the oocyte quality; which makes it a less direct measurement of the male partner contribution to a couple’s infertility. Conversely, damage to sperm chromatin organisation detected by sperm nuclear decondensation has little chance of being repaired by the oocyte/zygote and directly reflects the male contribution to infertility.

In summary, DFI and SDI are good indices of defective sperm quality but do not appear to be the best markers for the response to treatment so more specific tests are required. In our study chromatin decondensation index, which can be assessed either by aniline blue staining or by SCSA (HDS index), had the strongest correlation with outcome. To date it has been largely neglected.

The present study reports positive effects of dietary intervention on sperm damage indices and, possibly, on male fertility competence based upon a series of studies [3,5] on the same topic. In spite of the heterogeneity of design, assessment criteria and in the type and combinations of substances administered, all of these studies share a common aim: to readdress the oxidative imbalance. All point towards some positive effects; little doubt remains about the concept that sperm oxidative damage is a relevant clinical target and that it can be positively influenced by tailored nutritional supplementation. Nevertheless, the best possible interventional approach remains open to question and the present study may help elucidate this discussion.

The combination of substances administered to our patients had the potential to affect the sperm maturation process. Group B vitamins, which are cofactors for certain key metabolic enzymes, are available to the sperm but the amount is dependent on daily intake. The same applies to zinc. Moreover, zinc and vitamin B9 (folic acid), orally administered alone or in combination, have already been shown to positively influence sperm protamine content and acrosomal integrity [28]. N-acetyl-cysteine is soluble so it is easily absorbed and readily transformed into cysteine within the cell by the ubiquitous deacetylation reaction. Accordingly, oral administration of N-acetyl-cysteine was shown to improve semen parameters and the oxidative/antioxidant status in patients with male infertility [29]. Vitamin E is also readily available intracellularly and improved sperm motility as well as fertilising capacity when orally co-administered with zinc at a dose (20 mg per day) similar to that used in our study [30]. Finally, the importance of the availability of these substances from the diet and their effect on sperm function is confirmed by epidemiological findings. Men with the highest intake of vitamin E, folate, and zinc show less sperm DNA damage and older men with the highest intake of these micronutrients showed levels of sperm damage that were similar to those of younger men [31]. The combination of nutrients administered to our patients included substances which are either already widely used for nutritional support to spermatogenesis or had already been shown either alone or in combination to positively influence the redox balance.

From a chemical point of view the term antioxidant refers to compounds that prevent or delay the oxidation of other compounds; these include enzymatic (e.g. superoxide dismutase), non-enzymatic (e.g. vitamin C) and indirect antioxidants (e.g. chelating agents) [32]. The substances administered to our patients all belong to the class of indirect or very indirect antioxidants. The group B vitamins used are unable to directly reduce another substance; they only act as methyl donors or as co-enzymes for the one carbon cycle, i.e. they sustain transmethylations. In turn, full efficiency of Hcy remethylation provides amounts of SAMe that are essential for the activation of GSH synthesis, thereby raising antioxidant defence capacity. The same applies to zinc. Although formerly considered an antioxidant, N-acetyl-cysteine is also incapable of reducing other substances. It is, however, proven to act as a precursor for GSH synthesis and no further reducing effects can be demonstrated once GSH synthesis is saturated [33]. Betalains, which are provided in small amounts by our *opuntia* extract, have been demonstrated to be bioavailable and to selectively bind to lipids to prevent their oxidation, so reducing the burden of the lipoperoxidation cascade [34]. Quercetin (also from the *opuntia* extract) displays lipid membrane tropism whereby it inhibits the peroxidative cascade by chelating pro-oxidant metals [35]. Thus, betalain and quercetin contribute by reducing the burden of endogenous oxidative load from lipid peroxidation but do not directly induce a reductive environment. Finally, vitamin E is actively antioxidant at doses of 300 mg per day [36]: at the doses used in our study (12 mg per tablet) it can only exert mild protection on lipid peroxidation and cannot induce a reducing intracellular environment. Combined, these substances provide the necessary tools to push intracellular homeostasis towards fuller antioxidant capacity (GSH) while simultaneously supporting cellular growth and differentiation (one carbon cycle). This contrasts markedly with the artificial reducing environment produced by loading the system with direct antioxidants. A distinct added advantage of our approach is the minimization of the risk of induced “reductive” stress; precision of dosing is less critical as a result.

Our treatment (Condensyl™), is devoid of any direct antioxidants, apart from small amounts of vitamin E, and our results demonstrated a significant decrease of DFI, which is well known to be dependent on oxidative processes. These outcomes strongly support a primary role of the one-carbon cycle, in addition to its clear involvement in the transmethylations in redox regulation within the sperm cell. On the other hand, we could not investigate the effect of treatment on the global oxy-redox status of the patients and on the amount of oxidative DNA damage (e.g. 8-hydroxy-2′-deoxyguanosine levels), so a direct demonstration is still lacking. However, for the first time to the authors’ knowledge, and in direct contrast to the results obtained with strong oral antioxidants [8], the decrease of DFI paralleled a significant decrease of SDI, indicating that no reductive stress had occurred.

## Conclusions

The high degree of correlation of fertility improvement with the decrease of the decondensation index further supports the importance of the chromatin structure on the reproductive outcomes and its susceptibility to environmental factors, pollution and pro-oxidants on the one hand and dietary manipulation on the other. Sperm DNA damage indexes, DFI and SDI, confirm to mark a poor prognosis with SDI decrease exerting a robust relation with a positive outcome. Group B vitamins and their co-factors (Condensyl™) appear to be safe and effective and might improve the outcomes in both natural and assisted reproduction. Finally, a deeper investigation of the complex metabolic framework of human gametes has the potential to produce break-through understanding in the physiology of human reproduction and in its pathological conditions too.

## Abbreviations

ART: Assisted reproduction technologies; DFI: DNA fragmentation index; SDI: Sperm decondensation index; IUI: Intra uterine insemination; IVF: In vitro fertilization; ICSI: Intra cytoplasmic sperm injection; CPR: Clinical pregnancy rate; LBR: Live birth rate; GSH: Reduced glutathione; Hcy: Homocysteine; ROS: Reactive oxygen spedies; TUNEL: Terminal deoxynucleotidyl transferase dUTP Nick end labeling.

## Competing interests

DC, EA, MC and YM have no competing interests to disclose. MD is shareholder in Parthenogen, a private company engaged in the development of Condensyl.

## Authors’ contributions

YM, MC and MD conceived the study and designed the study procedures. DC, EA and MC conducted the clinical part of the study. MD and YM edited the manuscript. All authors read and approved the final manuscript.
